# KNTC1 and MCM2 are the molecular targets of gallbladder cancer

**DOI:** 10.18632/aging.204889

**Published:** 2023-07-21

**Authors:** Wei Jia, Chao Wang

**Affiliations:** 1 Department of Gastrointestinal Surgery, Beijing Rehabilitation Hospital, Capital Medical University, Badachu Xixia, Shijingshan 100144, Beijing, China; 2 Department of Hepatobiliary Surgery, The Fourth Affiliated Hospital of Hebei Medical University, Shijiazhuang 050011, Hebei, China

**Keywords:** KNTC1, MCM2, molecular targets, gallbladder cancer, bioinformatics

## Abstract

Background: Gallbladder carcinoma is a malignant epithelial tumor of gallbladder with a high degree of malignancy. However, relationship between KNTC1 and MCM2 and gallbladder cancer is unclear.

Methods: GSE139682 and GSE202479 were downloaded from gene expression omnibus (GEO). Differentially expressed genes (DEGs) were screened. Functional enrichment analysis and gene set enrichment analysis (GSEA) were performed. Protein-protein interaction (PPI) Network was constructed and analyzed. Gene expression heat map was drawn. Comparative toxicogenomics database (CTD) analysis was performed to find diseases most related to core genes. TargetScan was performed for screening miRNAs that regulated central DEGs.

Results: 230 DEGs were identified. According to GObp analysis, they were mainly concentrated in regulation of ossification, regulation of spindle microtubule and centromere attachment, cytoskeleton tissue of cortical actin. According to GOcc analysis, they are mainly concentrated in plasma membrane part, cell junction, plasma membrane region and anterior membrane. According to GOmf analysis, they are mainly enriched in protein homodimerization activity, proximal promoter sequence-specific DNA binding and sulfur compound binding. KEGG showed that target genes were mainly enriched in Hippo signal pathway, p53 signal pathway and cancer pathway. KIFC2, TUBG1, RACGAP1, CHMP4C, SFN and MYH11 were identified as core genes. Gene expression heat map showed that KNTC1, MCM2, CKAP2, RACGAP1, CCNB1 were highly expressed in gallbladder carcinoma samples. CTD analysis showed that KNTC1, MCM2, CKAP2, RACGAP1, CCNB1 were associated with head and neck squamous cell carcinoma, necrosis, inflammation and hepatomegaly.

Conclusions: KNTC1 and MCM2 are highly expressed in gallbladder cancer. Higher expression level correlates with worse prognosis.

## INTRODUCTION

Gallbladder carcinoma (GBC) is a kind of hepatobiliary malignant tumor developed from the intima of gallbladder mucosa [1, 2]. Although it is generally considered to be rare, it is the most common biliary malignant tumor, accounting for 80% Mel 95% of biliary cancer. The survival rate is low, which is mainly due to late diagnosis. Most symptomatic patients find incurable tumors with poor clinical outcomes [3]. The unique combination of inducing factors, including genetic susceptibility, geographical distribution, female bias, chronic inflammation and congenital dysplasia, makes this type of cancer unique. Epidemiological studies have found that there are amazing geographical and ethnic differences among American Indians. The incidence is very high in American Indians, high in Southeast Asia, but very low in the Americas and other parts of the world. Age, female, congenital biliary tract abnormality and genetic susceptibility are all important and unalterable risk factors [4]. The study found that incidence of gallbladder cancer in women is three to six times higher than men. In addition, incidence of gallbladder cancer continues to increase with age [5]. More than 2/3 of patients with gallbladder cancer were over 65 years old [6]. At present, the most effective treatment for GBC is surgery. However, due to the early asymptomatic characteristics and the concealment and rapid progression of the disease, a small number of patients are suitable for surgery. Chemotherapy, targeted therapy and immunotherapy are also used [7, 8]. However, because the cause of gallbladder cancer is not clear, the application of these treatments is still limited. Therefore, it is particularly important to study molecular mechanism of gallbladder cancer.

As an important part of the development of life science, bioinformatics has been at the forefront of life science and technology research. In recent years, China’s biotechnology has developed by leaps and bounds, and bioinformation resources have also grown explosively. Bioinformatics reveals the biological significance represented by big data, which is a bridge between data and clinic. Represented by the analysis and reporting of gene detection data, bioinformatics plays a role in the tumor treatment [9, 10].

However, the relationship between KNTC1, MCM2 genes and gallbladder cancer is not clear. Therefore, the paper intends to use the bioinformatics technology to explore and analyze core genes between gallbladder cancer and normal tissues. The public datasets were used to validate roles of KNTC1 and MCM2 in gallbladder cancer. And it was verified by basic cell experiment.

## RESULTS

### Screening of DEGs

**Figure 1:**
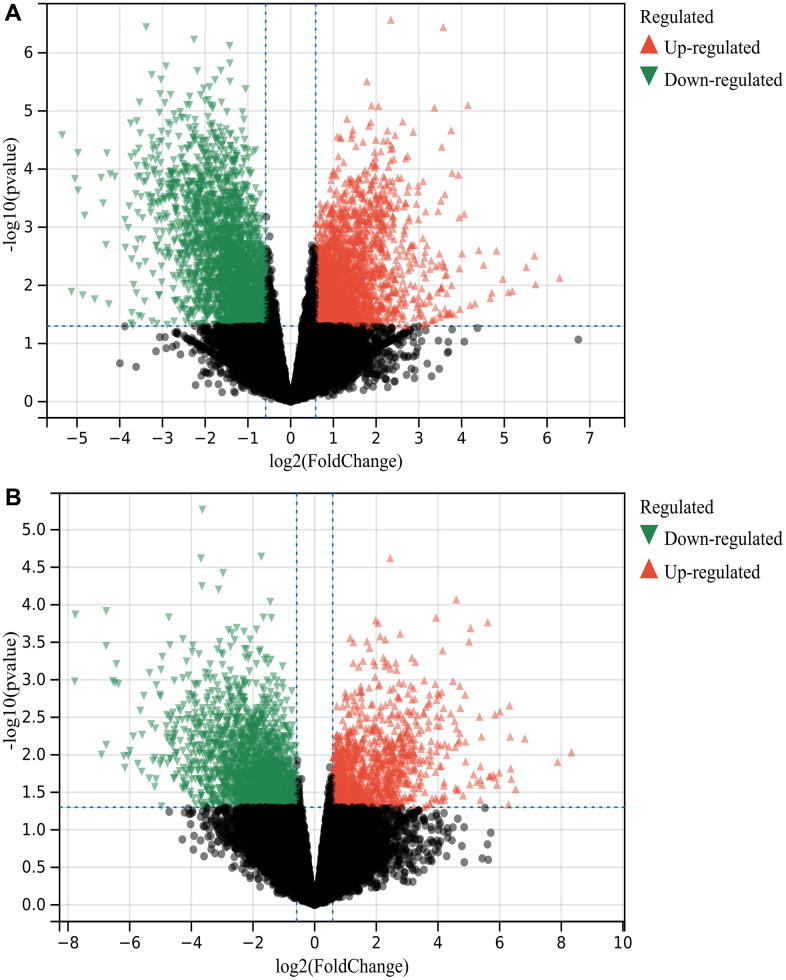
**Screening of DEGS.** 230 DEGs were identified (**A**) GSE139682 (**B**) GSE202479.

**Figure 2:**
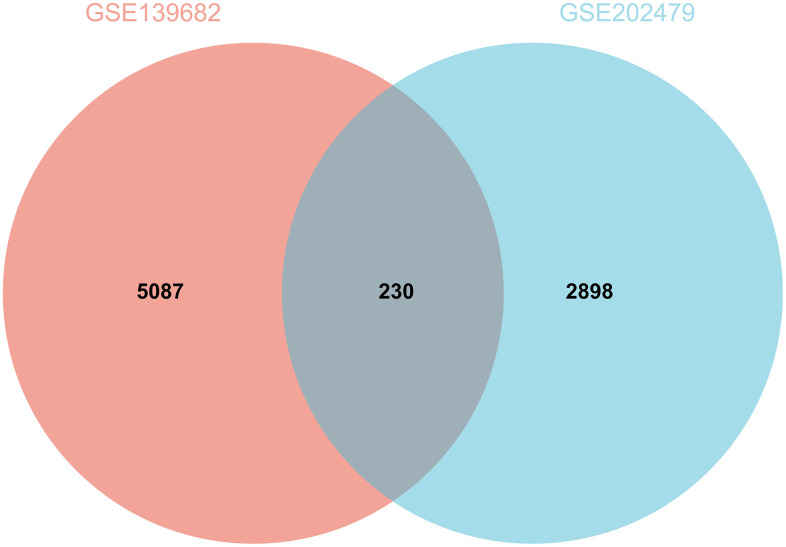
Screening of DEGs.

230 DEGs were identified according to the matrix of GSE139682 and GSE202479 (Figure 1A is the result of GSE139682, Figure 1B is the result of GSE202479, Figure 2).

### Functional enrichment analysis DEGs

**Figure 3:**
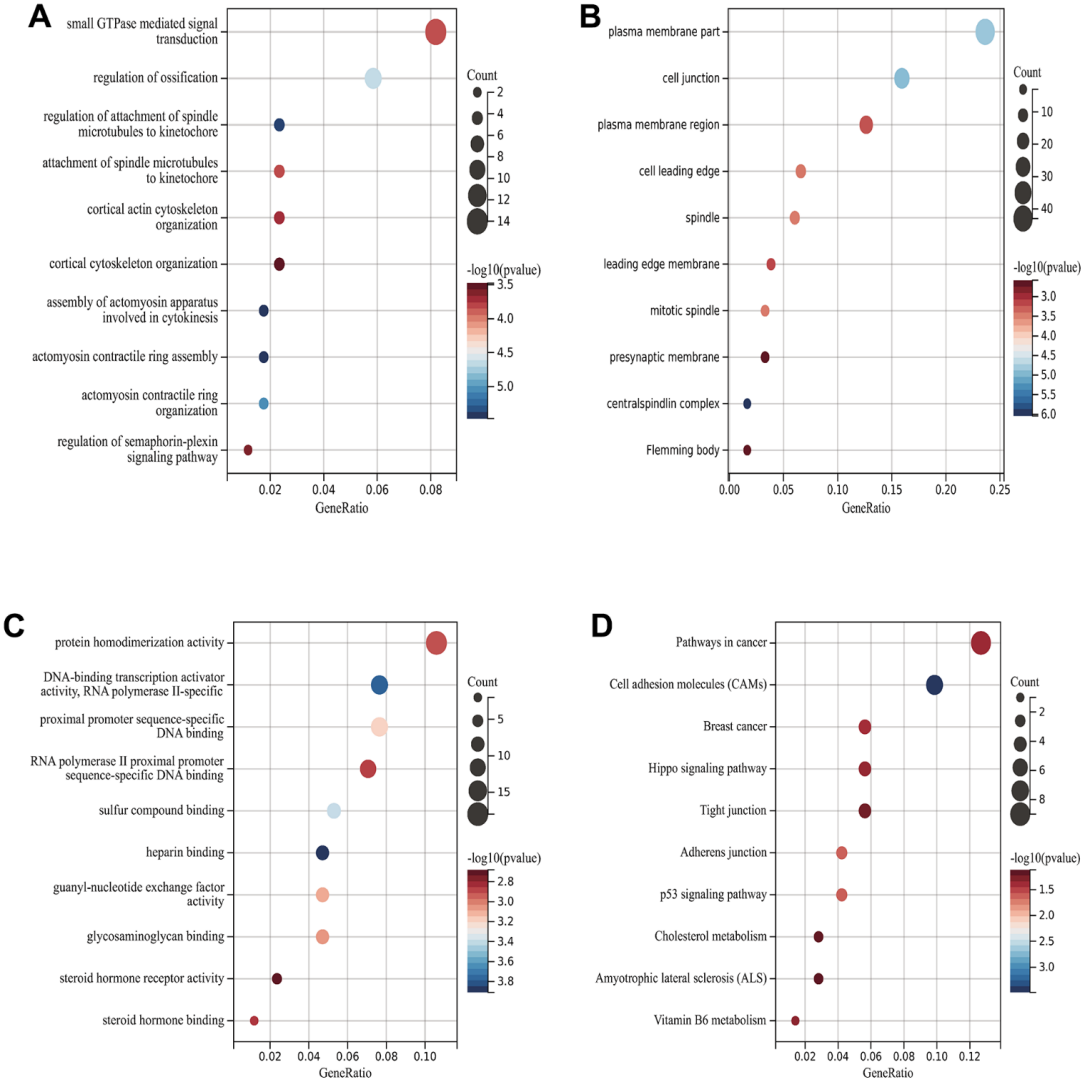
**Functional enrichment analysis of DEGs.** (**A**) GObp analysis (**B**) GOcc analysis (**C**) GOmf analysis (**D**) KEGG analysis.

We analyzed DEGs by GO and KEGG. According to GObp analysis, they were mainly concentrated in the regulation of ossification, the regulation of spindle microtubule and centromere attachment, and the cytoskeleton tissue of cortical actin (Figure 3A). According to GOcc analysis, they are mainly concentrated in the plasma membrane part, cell junction, plasma membrane region and anterior membrane (Figure 3B). According to GOmf analysis, they are mainly enriched in protein homodimerization activity, proximal promoter sequence-specific DNA binding and sulfur compound binding (Figure 3C).

KEGG analysis showed that target genes were mainly enriched in Hippo signal pathway, p53 signal pathway and cancer pathway (Figure 3D).

### GSEA

**Figure 4:**
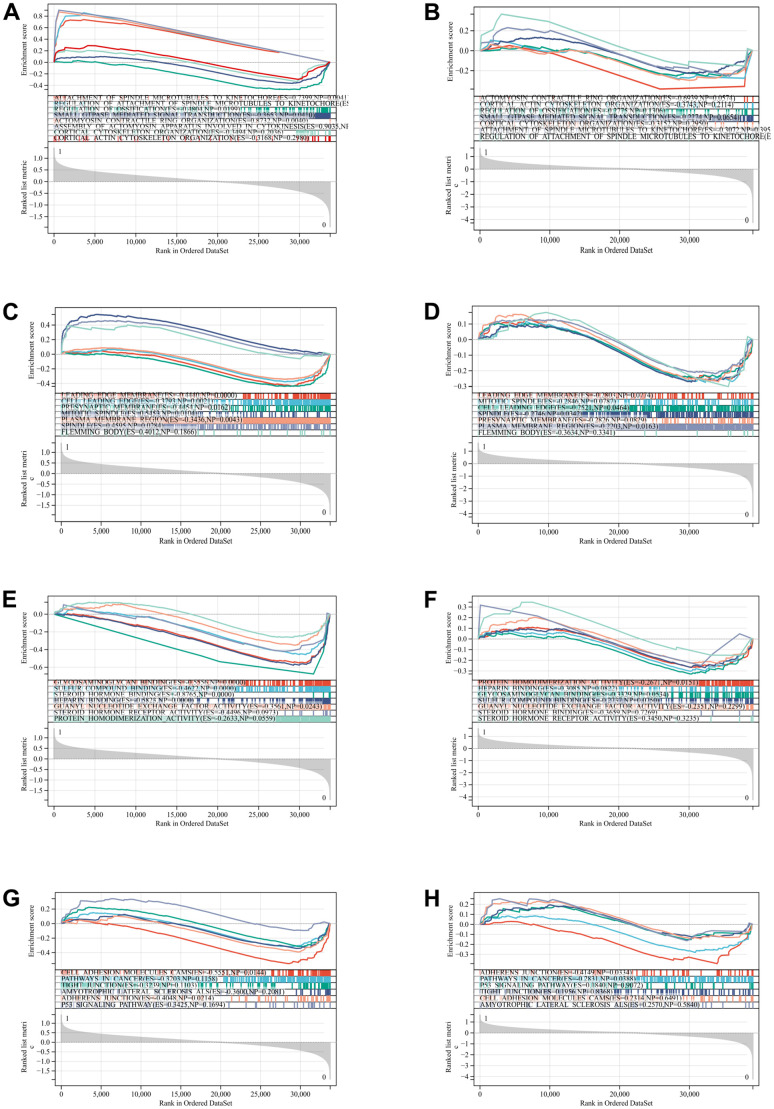
**GSEA for the DEGs.** (**A**) Attachment of spindle microtubules. (**B**) Actomyosin contractile ring organization. (**C**) Leading edge membrane. (**D**) Mitotic spindle. (**E**) Glycosaminoglycan binding. (**F**) Protein homodimerization activity. (**G**) Cell adhesion molecules cams. (**H**) Adherens junction.

GSEA was performed to search for possible enrichment items among non-differentially expressed genes, and results of DEGs were verified. The intersection of enrichment items and GOKEGG enrichment items of differentially expressed genes is shown in the figure, which is mainly concentrated in Hippo signal pathway, p53 signal pathway and cancer pathway. (Figure 4A, 4C, 4E, 4G are GSE139682 results, while Figure 4B, 4D, 4F, 4H are GSE202479 results).

### Metascape enrichment analysis

**Figure 5:**
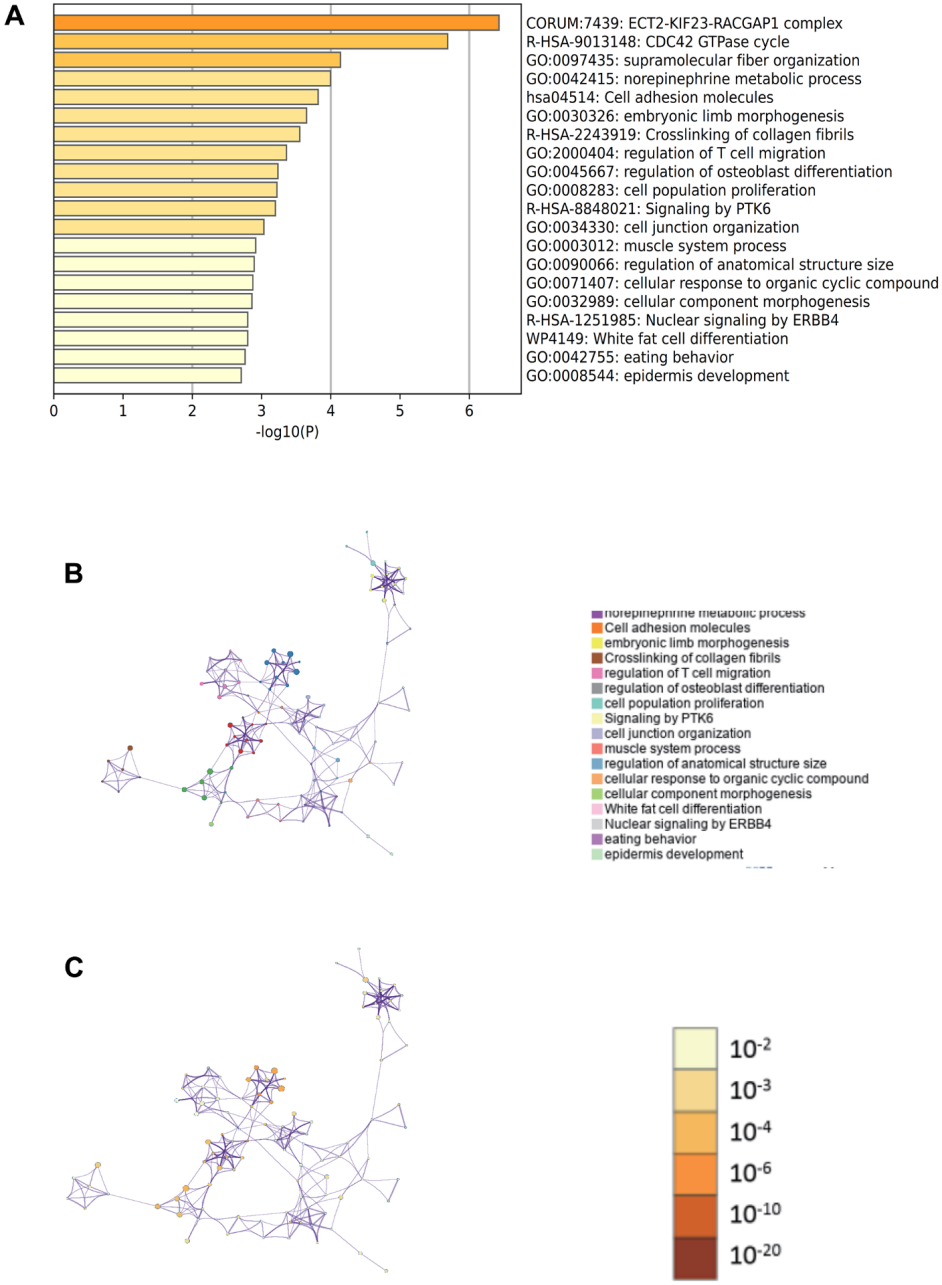
**Metascape enrichment analysis.** (**A**) GO has the regulation of supramolecular fibrous tissue, norepinephrine metabolism and T cell migration. (**B**) Enrichment networks colored by enrichment terms. (**C**) Enrichment networks colored by p values.

**Figure 6:**
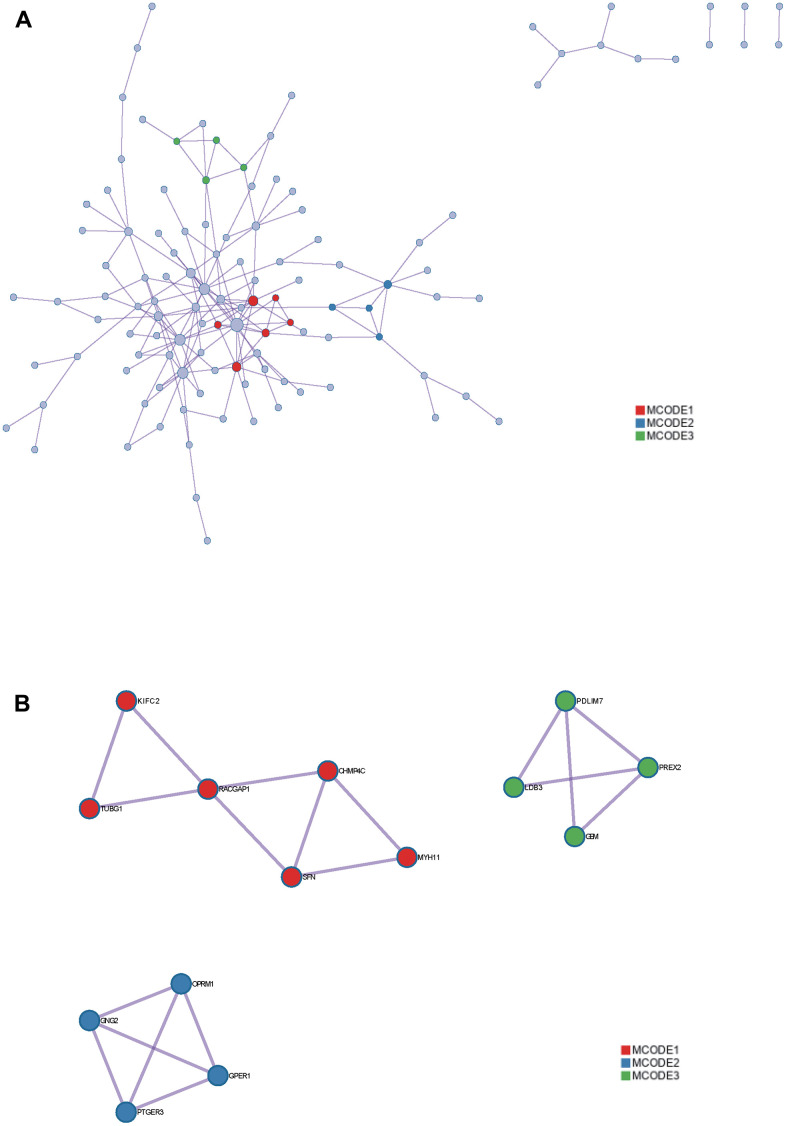
**Metascape enrichment analysis.** Red: MCODE1. Blue: MCODE2. Green: MCODE3. (**A**) protein-protein interact among the DEGs by the Metascape. (**B**) MCODE in the network.

In the enrichment project of Metascape, GO has the regulation of supramolecular fibrous tissue, norepinephrine metabolism and T cell migration (Figure 5A), and an enrichment network stained by enrichment term and p-value (Figures 5B, 5C, 6), which visually shows the correlation and confidence of each enrichment item.

### PPI network

**Figure 7:**
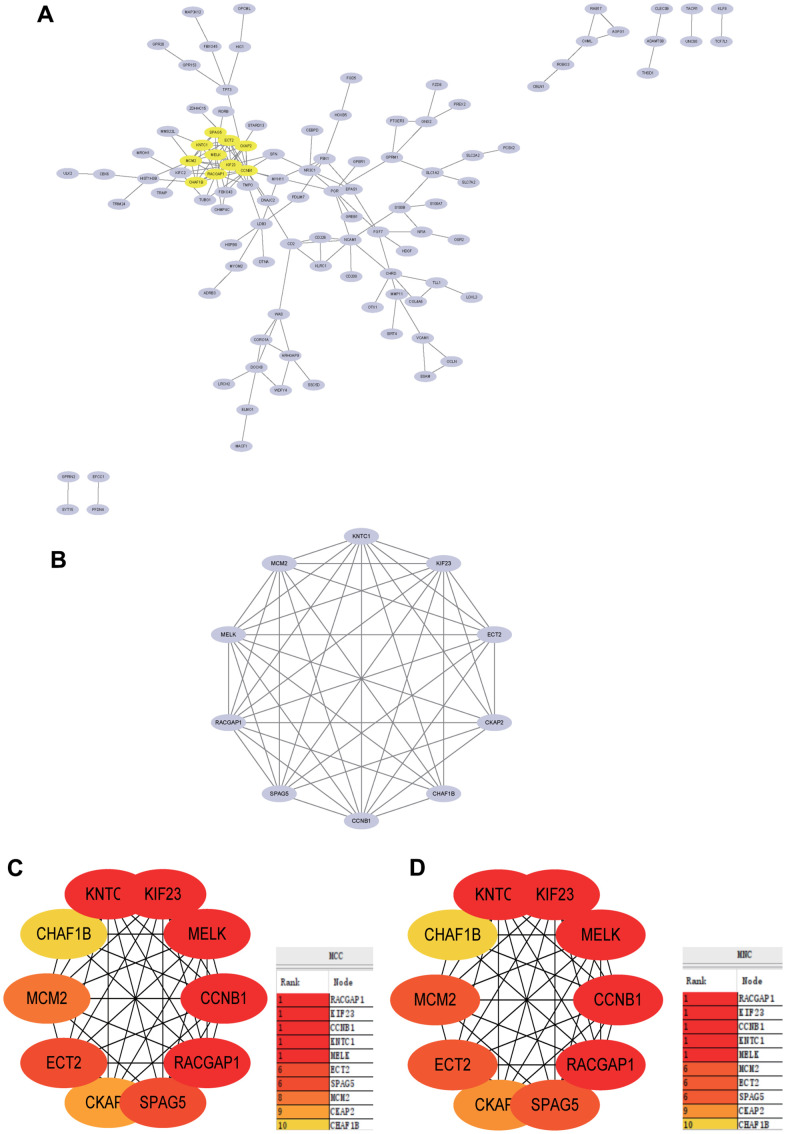
**Construction and analysis of protein-protein interaction (PPI) network.** (**A**) PPI network. (**B**) The core gene cluster. (**C**) MCC was used to identify central genes. (**D**) MNC was used to identify central genes.

**Figure 8:**
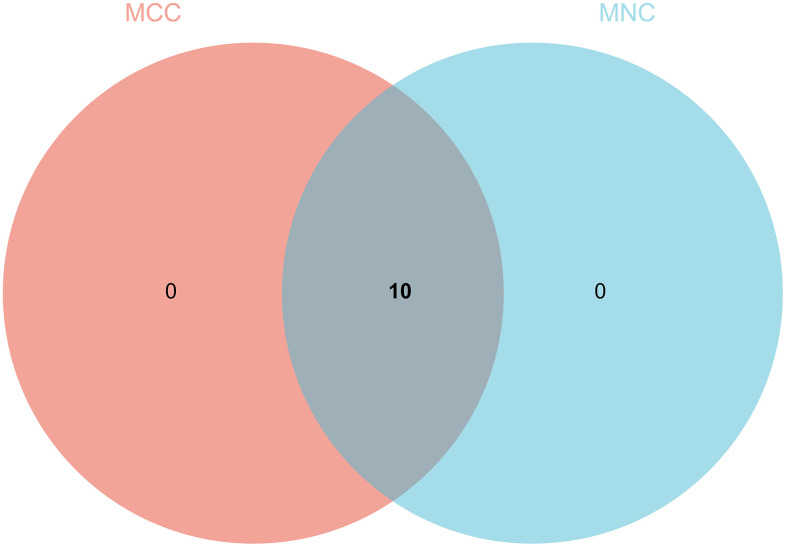
**Wayne diagram and intersection.** 10 core genes (ECT2L, MELK, SPAG5, KIF23, CHAF1B, KNTC1, MCM2, CKAP2, RACGAP1, CCNB1) are obtained.

The PPI network was constructed by STRING and analyzed by Cytoscape (Figure 7A), the core gene cluster is obtained (Figure 7B). The hub genes were identified using two different algorithms (Figure 7C, 7D), Wayne diagram and intersection (Figure 8) are drawn, and 10 core genes (ECT2L, MELK, SPAG5, KIF23, CHAF1B, KNTC1, MCM2, CKAP2, RACGAP1, CCNB1) are obtained.

At the same time, we also use the Metascape website to output the protein interaction network, and identify the core module to verify the PPI network results in STRING. Among them, KIFC2, TUBG1, RACGAP1, CHMP4C, SFN and MYH11 genes were identified as core genes.

### Gene expression heat map

**Figure 9:**
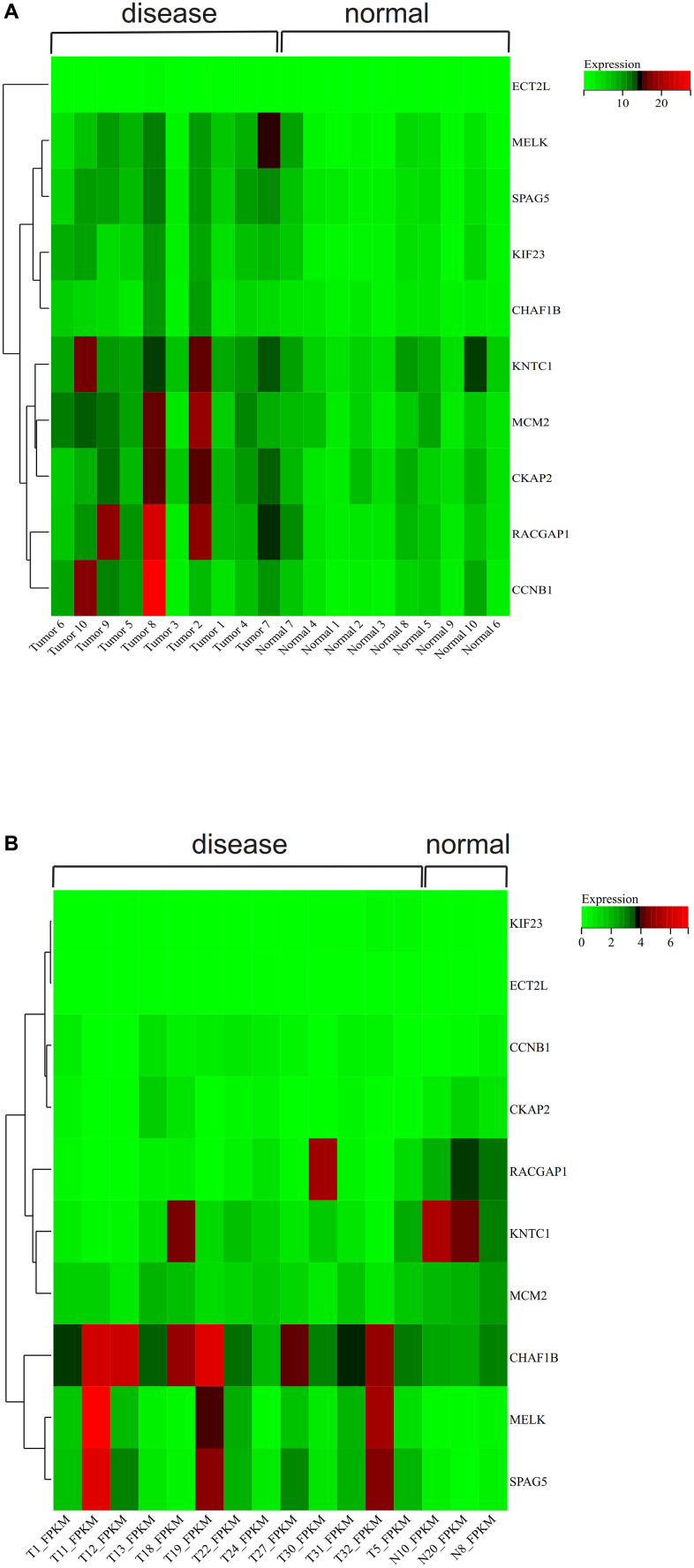
**>Gene expression heat map.** (**A**) GSE139682 and (**B**) GSE202479.

We obtained a visual differential heat map of core genes between gallbladder carcinoma and normal samples. We found that five core genes (KNTC1, MCM2, CKAP2, RACGAP1, CCNB1) were highly expressed in gallbladder carcinoma samples and low in normal samples. ECT2L, MELK, SPAG5, KIF23 and CHAF1B genes may play a regulatory role in gallbladder carcinoma (Figure 9A is the result of GSE139682, Figure 9B is the result of GSE202479).

### CTD analysis

**Figure 10:**
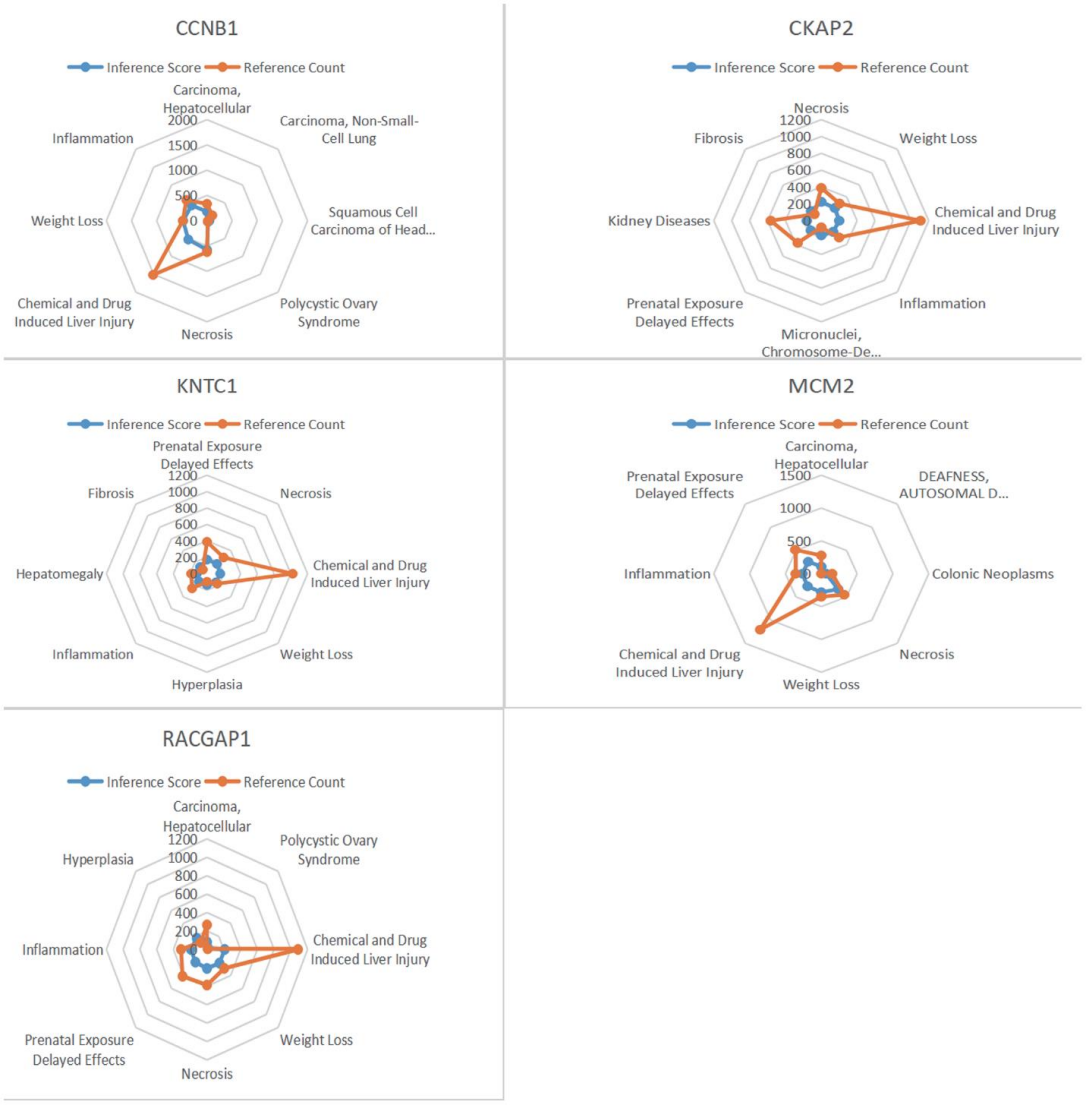
**CTD analysis.** Five genes (KNTC1, MCM2, CKAP2, RACGAP1, CCNB1) were found to be associated with head and neck squamous cell carcinoma, necrosis, inflammation and hepatomegaly.

Core genes were entered into CTD to find diseases related to core genes. Five genes (KNTC1, MCM2, CKAP2, RACGAP1, CCNB1) were found to be associated with head and neck squamous cell carcinoma, necrosis, inflammation and hepatomegaly (Figure 10).

### The miRNA prediction and functional annotation related to hub gene

Hub genes were input into TargetScan to search for related miRNAs (Table 1). The related miRNA of ECT2L gene is the correlation of hsa-miR-4262, hsa-miR-181c-5p and hsa-miR-181b-5p; MELK genes. Mirna is the correlation of hsa-miR-802; KIF23 gene, miRNA is hsa-miR-107, miRNA of hsa-miR103a-3p; CHAF1B gene is hsa-miR-216a-5p; KNTC1 gene, miRNA is hsa-miR-138-5p, miRNA of RACGAP1 gene is hsa-miR-19b-3p, hsamiR-19a-3p. The related miRNA of CCNB1 gene is hsa-miR-183-5p.1.

**Table 1:** A summary of miRNAs that regulate hub genes.

	**Gene**	**MIRNA**		
**1**	**ECT2L**	hsa-miR-4262	hsa-miR-181c-5p	hsa-miR-181b-5p
**2**	**MELK**	hsa-miR-802		
**3**	**SPAG5**	none		
**4**	**KIF23**	hsa-miR-107	hsa-miR-103a-3p	
**5**	**CHAF1B**	hsa-miR-216a-5p		
**6**	**KNTC1**	hsa-miR-138-5p		
**7**	**MCM2**	none		
**8**	**CKAP2**	none		
**9**	**RACGAP1**	hsa-miR-19b-3p	hsa-miR-19a-3p	
**10**	**CCNB1**	hsa-miR-183-5p.1		

## DISCUSSION

Gallbladder cancer is a highly malignant disease with poor prognosis, high invasion and metastasis rate and mortality. GBC is the most invasive biliary tract cancer with the shortest median survival time [11, 12]. The available treatment options vary widely in areas with a high prevalence of gallbladder cancer, resulting in different outcomes for patients in different regions. Although treated in the most advanced areas of medicine, malignant tumors of gallbladder are highly fatal. Only about 1/5 of gallbladder cancer cases are found when the disease is still confined to the gallbladder, according to the American Cancer Society. This greatly limits the choice of therapeutic treatment and reduces the overall survival rate [8, 13, 14]. The exact molecular mechanism of gallbladder cancer is not clear. Although many studies have reported key genes related to gallbladder cancer, few studies have proved that these loci have been well applied to the diagnosis and treatment of gallbladder cancer. Many genes and molecules play a key role in pathophysiological processes, and understanding their interactions is important for the molecular mechanism of gallbladder cancera [15]. At present, many bioinformatics techniques can explore the intratumoral heterogeneity and cancer progression. The main result of this study is that KNTC1 and MCM2 genes are highly expressed in gallbladder carcinoma. The higher the KNTC1 and MCM2 genes are, the worse the prognosis is.

KNTC1 encodes a mitochondrial component in the Rod-Zwilch-ZW10 (RZZ) complex, which is the key to sister chromatid separation and participation in spindle checkpoints during mitosis. Kinetochore-associated proteins are key components of mitotic checkpoints and are essential for faithful chromosome separation and spindle assembly during cell division [16–18]. Recent studies have shown that KNTC1 may be a potential biomarker for promoting the occurrence and development of human malignant tumors [19]. Chromosome segregation and cell division are key biological processes, in which many evolutionarily conserved protein complexes. These proteins are overexpressed in malignant tumors, and some of them have even been identified as oncogenes [20]. Recent advances have shown that kinetochore-associated proteins are up-regulated and play a role in carcinogenesis of many types of cancer. The literature shows that KNTC1 may affect the biological activity of hepatocellular carcinoma cells through PI3K/Akt signal pathway, and high KNTC1 expression is associated with the poor prognosis [19]. KNTC1 can be used as a tumor promoter in the occurrence and development of non-small cell lung cancer by colony formation, cell migration and inhibiting apoptosis. It is worth noting that KNTC1 may regulate non-small cell lung cancer through its downstream target PSMB8 [21]. In addition, it is reported that KNTC1 is associated with esophageal squamous cell carcinoma. The down-regulation of KNTC1 expression inhibits cell viability and induces apoptosis in the ESCC cell line [22]. Zhang et al. found that KNTC1 was up-regulated in colon cancer compared with normal tissues. The high KNTC1 expression was associated with the poor prognosis [18]. These studies suggest that kinetin can be used as a potential biomarker for early diagnosis of cancer. Similarly, we also found that KNTC1 gene is highly expressed in gallbladder cancer, higher the KNTC1, worse the prognosis.

MCM2 encodes a 904 amino acid protein with a molecular weight of 101896Da. MCM2 is a member of microchromosome maintenance protein family, regulates DNA replication and cell cycle by participating in formation of replication initiation complex [23–25]. Previous studies have shown that inhibition of MCM2 reduces cell viability and aggravates apoptosis in cellular models of Alzheimer’s disease [26]. The MCM2 protein is overexpressed in the nucleus of high malignant tumors, which is related to the late stage, late stage and poor prognosis of the tumor. Gulinisha Aihemaiti found that MCM2 was highly expressed in the ovarian clear cell carcinoma, and the expression of MCM2 was mainly confined to nucleus [27]. In addition, Deng et al. reported that knockdown of MCM2 can improve chemoresistance of ovarian cancer to carboplatin and olaparib [28]. A study of gastric cancer reported that CAMKK2 can over-activate microchromosome maintenance proteins in gastric cancer cells through MEK/ERK pathway to promote cancer cell proliferation [29]. Similarly, in our study, we also found that the MCM2 gene is highly expressed in gallbladder cancer, and higher the MCM2, worse the prognosis. This is consistent with results of previous studies, which speculated that MCM2 may play a role in progression of gallbladder cancer.

Although this paper has carried out rigorous bioinformatics analysis, there are still some shortcomings. Animal experiments with overexpression or knockdown of the gene were not performed in this study to further verify its function. Therefore, this aspect should be explored in depth in future studies.

To sum up, KNTC1 and MCM2 are highly expressed in gallbladder carcinoma, and may play a role in occurrence and development of gallbladder carcinoma through many ways. KNTC1 and MCM2 may be molecular targets for early diagnosis and precise treatment of gallbladder cancer, and provide a basis for the study of the mechanism of gallbladder cancer.

## MATERIALS AND METHODS

### Gallbladder cancer data set

The gallbladder cancer dataset GSE139682 and GSE202479 were downloaded from GEO database generated by GPL20795 and GPL24676. GSE139682 included 10 gallbladder carcinomas and 10 normal samples, GSE202479 included 13 gallbladder carcinomas and 3 normal samples. It is used to identify the DEGs in gallbladder carcinoma.

### Screening of DEGs

Probe aggregation and background correction of merge matrix of GSE139682 and GSE202479 using R package “limma”. P value were adjusted using Benjamini-Hochberg method. The fold change (FC) is calculated using false discovery rate (FDR). The cutoff value of DEG is p less than 0.05 and FC is greater than 1.5. And make a visual representation of the volcano, and the intersection DEG of GSE139682 and GSE202479 is obtained by Wayne diagram.

### Functional enrichment analysis

Gene Ontology (GO) and Kyoto Encyclopedia of Gene and Genome (KEGG) are computational methods for evaluating function and biological pathways of genetics. Differential genes screened by Wayne map was input into KEGG rest API obtained latest KEGG Pathway gene annotation, which was used as background. Gene set enrichment results were obtained using R package cluster Profiler.

Metascape (http://metascape.org/) is a gene function annotation and analysis tool that can realize the cognition of gene or protein function, and can be visually exported. We used Metascape to analyze functional enrichment of above differential gene list and derive it.

### GSEA

GSEA is based on level-specific gene probes that evaluate data from microarrays and is a way to uncover genomic expression data through fundamental knowledge. According to degree of carotid atherosclerosis, samples were divided into normal sample and carotid atherosclerosis group. Relevant pathways and molecular mechanisms were evaluated. 5 is minimum gene set and 5000 is maximum gene set, and 1000 resampling times. The whole genome was analyzed by GO and KEGG. Developed by GSEA.

### Construction and analysis of protein-protein interaction (PPI) network

Search Tool for the Retrieval of Interacting Genes (STRING) is a search system for known and predicted PPI. STRING database also contains the predicted results using bioinformatics methods. The differential genes were input into STRING to construct PPI network and predict the core genes. PPI network was visualized, core genes are predicted by Cytoscape software. First of all, we import PPI network into the Cytoscape, and then genes with best correlation were calculated by MCC and MNC. Finally, core genes were obtained after visualization.

### Gene expression heat map

The expression of core genes in GSE139682 and GSE202479 PPI networks was mapped using the R-packet heat map, and to visualize difference of core gene expression between gallbladder cancer and normal samples.

### CTD analysis

CTD provides manually curated information on chemo-gene/protein interactions, gene-disease relationships, is a powerful public database. The core genes were input into CTD, so as to find the diseases most related to core gene. Excel was used to draw radar map of differential expression of each gene.

### The miRNA

TargetScan (https://www.targetscan.org) can predict and analyze miRNA and target genes. Screening of miRNAs regulating central DEGs was performed using TargetScan in this study.

### Data availability

The datasets generated during and/or analyzed during the current study are available from the corresponding author on reasonable request.

## References

[1] Sturm N, Schuhbaur JS, Hüttner F, Perkhofer L, Ettrich TJ. Gallbladder Cancer: Current Multimodality Treatment Concepts and Future Directions. Cancers (Basel). 2022; 14:5580. 10.3390/cancers14225580 36428670 PMC9688543

[2] Krell RW, Wei AC. Gallbladder cancer: surgical management. Chin Clin Oncol. 2019; 8:36. 10.21037/cco.2019.06.06 31431029

[3] PDQ Adult Treatment Editorial Board. Gallbladder Cancer Treatment (PDQ®): Patient Version. 2023. In: PDQ Cancer Information Summaries. Bethesda (MD): National Cancer Institute (US). 2002.

[4] Malik MA, Malik SA, Haq MG, Bangri SA, Ahmad SZ, Shah OJ, Shah ZA. Genetic Susceptibility of DCC Gene in Gallbladder Cancer in Kashmir and Meta-Analysis. Nutr Cancer. 2022; 74:947–55. 10.1080/01635581.2021.1949728 34259111

[5] Schmidt MA, Marcano-Bonilla L, Roberts LR. Gallbladder cancer: epidemiology and genetic risk associations. Chin Clin Oncol. 2019; 8:31. 10.21037/cco.2019.08.13 31484487

[6] Mao W, Deng F, Wang D, Gao L, Shi X. Treatment of advanced gallbladder cancer: A SEER-based study. Cancer Med. 2020; 9:141–50. 10.1002/cam4.2679 31721465 PMC6943088

[7] Baiu I, Visser B. Gallbladder Cancer. JAMA. 2018; 320:1294. 10.1001/jama.2018.11815 30264121

[8] Gallbladder cancer. Nat Rev Dis Primers. 2022; 8:70. 10.1038/s41572-022-00403-4 36302789 PMC12314663

[9] Shao T, Xie Y, Shi J, Yang C, Zou H, Li Y, Xu J, Li X. Surveying lncRNA-lncRNA cooperations reveals dominant effect on tumor immunity cross cancers. Commun Biol. 2022; 5:1324. 10.1038/s42003-022-04249-0 36463330 PMC9719535

[10] Goh JJ, Goh CJ, Lim QW, Zhang S, Koh CG, Chiam KH. Transcriptomics indicate nuclear division and cell adhesion not recapitulated in MCF7 and MCF10A compared to luminal A breast tumours. Sci Rep. 2022; 12:20902. 10.1038/s41598-022-24511-z 36463288 PMC9719475

[11] Mukkamalla SKR, Kashyap S, Recio-Boiles A, Babiker HM. Gallbladder Cancer. In: StatPearls. Treasure Island (FL): StatPearls Publishing. 2023.

[12] Roa JC, García P, Kapoor VK, Maithel SK, Javle M, Koshiol J. Publisher Correction: Gallbladder cancer. Nat Rev Dis Primers. 2022; 8:75. 10.1038/s41572-022-00408-z 36400795

[13] Sharma A, Sharma KL, Gupta A, Yadav A, Kumar A. Gallbladder cancer epidemiology, pathogenesis and molecular genetics: Recent update. World J Gastroenterol. 2017; 23:3978–98. 10.3748/wjg.v23.i22.3978 28652652 PMC5473118

[14] Hickman L, Contreras C. Gallbladder Cancer: Diagnosis, Surgical Management, and Adjuvant Therapies. Surg Clin North Am. 2019; 99:337–55. 10.1016/j.suc.2018.12.008 30846038

[15] Chen P, Wang Y, Li J, Bo X, Wang J, Nan L, Wang C, Ba Q, Liu H, Wang H. Diversity and intratumoral heterogeneity in human gallbladder cancer progression revealed by single-cell RNA sequencing. Clin Transl Med. 2021; 11:e462. 10.1002/ctm2.462 34185421 PMC8236117

[16] Wang C, Wang Y, Liu C, Meng X, Hang Z. Kinetochore-associated protein 1 promotes the invasion and tumorigenicity of cervical cancer cells via matrix metalloproteinase-2 and matrix metalloproteinase-9. Bioengineered. 2022; 13:9495–507. 10.1080/21655979.2022.2061144 35389773 PMC9161993

[17] Xu Z, Wang S, Ren Z, Gao X, Xu L, Zhang S, Ren B. An integrated analysis of prognostic and immune infiltrates for hub genes as potential survival indicators in patients with lung adenocarcinoma. World J Surg Oncol. 2022; 20:99. 10.1186/s12957-022-02543-z 35354488 PMC8966338

[18] Zhengxiang Z, Yunxiang T, Zhiping L, Zhimin Y. KNTC1 knockdown suppresses cell proliferation of colon cancer. 3 Biotech. 2021; 11:262. 10.1007/s13205-021-02800-0 33996374 PMC8113418

[19] Tong H, Liu X, Peng C, Shen B, Zhu Z. Silencing of KNTC1 inhibits hepatocellular carcinoma cells progression via suppressing PI3K/Akt pathway. Cell Signal. 2023; 101:110498. 10.1016/j.cellsig.2022.110498 36273753

[20] Chan GK, Jablonski SA, Starr DA, Goldberg ML, Yen TJ. Human Zw10 and ROD are mitotic checkpoint proteins that bind to kinetochores. Nat Cell Biol. 2000; 2:944–7. 10.1038/35046598 11146660

[21] Liu R, Liu R, Guo Z, Ren J, Huang J, Luo Q, Tan Q. shRNA-mediated knockdown of KNTC1 inhibits non-small-cell lung cancer through regulating PSMB8. Cell Death Dis. 2022; 13:685. 10.1038/s41419-022-05140-w 35933405 PMC9357013

[22] Liu CT, Min L, Wang YJ, Li P, Wu YD, Zhang ST. shRNA-mediated knockdown of KNTC1 suppresses cell viability and induces apoptosis in esophageal squamous cell carcinoma. Int J Oncol. 2019; 54:1053–60. 10.3892/ijo.2019.4672 30628654

[23] Guerrero-Puigdevall M, Fernandez-Fuentes N, Frigola J. Stabilisation of half MCM ring by Cdt1 during DNA insertion. Nat Commun. 2021; 12:1746. 10.1038/s41467-021-21932-8 33741931 PMC7979726

[24] Zhai Y, Li N, Jiang H, Huang X, Gao N, Tye BK. Unique Roles of the Non-identical MCM Subunits in DNA Replication Licensing. Mol Cell. 2017; 67:168–79. 10.1016/j.molcel.2017.06.016 28732205

[25] Yuan J, Lan H, Huang D, Guo X, Liu C, Liu S, Zhang P, Cheng Y, Xiao S. Multi-Omics Analysis of MCM2 as a Promising Biomarker in Pan-Cancer. Front Cell Dev Biol. 2022; 10:852135. 10.3389/fcell.2022.852135 35693940 PMC9174984

[26] Guan F, Gao Q, Dai X, Li L, Bao R, Gu J. LncRNA RP11-59J16.2 aggravates apoptosis and increases tau phosphorylation by targeting MCM2 in AD. Front Genet. 2022; 13:824495. 10.3389/fgene.2022.824495 36092938 PMC9459667

[27] Aihemaiti G, Kurata M, Nogawa D, Yamamoto A, Mineo T, Onishi I, Kinowaki Y, Jin XH, Tatsuzawa A, Miyasaka N, Kitagawa M, Yamamoto K. Subcellular localization of MCM2 correlates with the prognosis of ovarian clear cell carcinoma. Oncotarget. 2018; 9:28213–25. 10.18632/oncotarget.25613 29963273 PMC6021330

[28] Deng M, Sun J, Xie S, Zhen H, Wang Y, Zhong A, Zhang H, Lu R, Guo L. Inhibition of MCM2 enhances the sensitivity of ovarian cancer cell to carboplatin. Mol Med Rep. 2019; 20:2258–66. 10.3892/mmr.2019.10477 31322224 PMC6691261

[29] Najar MA, Aravind A, Dagamajalu S, Sidransky D, Ashktorab H, Smoot DT, Gowda H, Prasad TSK, Modi PK, Chatterjee A. Hyperactivation of MEK/ERK pathway by Ca2+ /calmodulin-dependent protein kinase kinase 2 promotes cellular proliferation by activating cyclin-dependent kinases and minichromosome maintenance protein in gastric cancer cells. Mol Carcinog. 2021; 60:769–83. 10.1002/mc.23343 34437731

